# Characterization of postoperative LASIK ectasia features on higher-order aberration excimer ablation maps

**DOI:** 10.1186/s12886-023-03263-y

**Published:** 2023-12-20

**Authors:** Avi Wallerstein, Sangeetha Santhakumaran, Lauren Tabunar, Mark Cohen, Mathieu Gauvin

**Affiliations:** 1 LASIK MD, 1250 Rene-Levesque Blvd W, MD Level, H3B 4W8 Montreal, QC Canada; 2 Department of Ophthalmology and Visual Sciences, McGill University, Montreal, QC Canada; 3 Department of Ophthalmology, Queen’s University, Kingston, ON Canada; 4 Department of Surgery, University of Sherbrooke, Sherbrooke, QC Canada

**Keywords:** Topography, Contoura, Ectasia, Diagnosis, Higher-order aberration

## Abstract

**Background:**

To characterize anterior corneal higher-order aberration (HOA) excimer ablation map patterns in postoperative LASIK ectasia (POE) and to examine correlations between newly identified corneal HOA ablation map features of POE and known topographic indices.

**Methods:**

Prospective multicenter non-interventional descriptive study. A total of 28 eyes from 22 POE patients were enrolled. The postoperative HOA ablation map was derived from Topolyzer Vario corneal imaging at the time of POE diagnosis. Features that recurred were identified and then analyzed. Correlations to Orbscan indices were studied.

**Results:**

An arrangement of two elliptical paracentral ablation islands, deep inferior and shallow superior, in direct mirror-like opposition to each other, were identified on all HOA maps. The paracentral islands were accompanied by peripheral ablation crescents. The deep paracentral inferior island ‘hot spot’ coincided with the topographical apical POE cone and was highly reproducible in angular position (249.3 ± 17.3°). There was significant variation in ablation depth (shallow superior island: 11.5 ± 6.9 μm and deep inferior island: 32.5 ± 18.8 μm). The superior crescents had high variability in depth (34.8 ± 18.9 μm). Strong correlations were found between the corneal irregularity index and the ablation depth difference between the deep and shallow paracentral islands (R = 0.96; *P* < 0.0001).

**Conclusion:**

The corneal HOA excimer ablation map revealed a recurring, distinct, easily recognizable pattern in POE eyes. Validated Orbscan POE indices and HOA ablation map islands showed a strong correlation. It is possible to extract useful information from the corneal HOA ablation map, potentially making it suitable for diagnosing and monitoring POE although more studies are needed.

**Supplementary Information:**

The online version contains supplementary material available at 10.1186/s12886-023-03263-y.

## Background

Postoperative ectasia (POE) is a rare adverse outcome of laser in-situ keratomileusis (LASIK) refractive surgery. The reported prevalence of POE is low, ranging between 0.01% and 0.6% [1, 2]. POE is characterized by reduced corneal biomechanical stability, progressive corneal steepening, stromal thinning, and irregular astigmatism [3, 4]. With time, these changes result in visual impairment and associated patient morbidity. Diagnosis of POE is made using clinical findings coupled with topographic, topometric, tomographic, and biomechanical indices [2, 5]. While the ultimate diagnostic challenge lies in predicting the risk of POE preoperatively, identifying early-stage POE is equally important for timely intervention with corneal crosslinking [6, 7].

Indicators to diagnose POE and keratoconus are similar, including the use of corneal anterior and posterior elevation and curvature, corneal thickness and biomechanical indices, epithelial patterns, and wavefront aberration metrics [8. 9]. Currently, no single diagnostic parameter is sensitive enough to distinguish early cases of POE from normals. Indices from multiple corneal imaging modalities are generally used to guide decision-making.

The Alcon platform for topography-guided excimer treatments uses the WaveLight ® Topolyzer™ VARIO high-resolution Placido disc topographer to image the cornea and create HOA ablation maps preoperatively. The topographic data is used by the excimer laser treatment software (named Contoura) to generate a treatment ablation map that combines both lower-order aberration astigmatism and higher-order aberration (HOA) data into one image, providing ablation depth data [8]. The Contoura planning software allows one to separate out and exclusively see the anterior corneal HOA ablation map by setting the sphere and cylinder treatments to zero. This map graphically shows the location and depth of anterior corneal HOAs to be treated with the excimer laser [8–11]. The Topolyzer™ VARIO and resulting HOA ablation maps are used for treatment and have never been used as a POE diagnostic tool. While corneal topography provides valuable information about the shape and curvature of the cornea, this map provides complementary details, specifically highlighting localized corneal higher-order aberrations that may not be fully captured by corneal topography alone.

Despite previously being reported in virgin corneas and keratoconus [8, 12], eyes with POE have not been investigated for corneal HOA ablation map patterns or ablation depths. The current study aims to thoroughly characterize the corneal HOA ablation map patterns in POE eyes and determine if a correlation exists between newly identified POE corneal HOA ablation map features and other gold-standard topographic indices of POE, such as maximal keratometry, corneal irregularity indices, and posterior elevation. This paper’s goal is to first characterize the HOA ablation map in POE eyes as a first step for future studies to validate the inferiority or superiority of the HOA ablation map in identifying or grading POE.

## Methods

### Selection of POE patients

The study was a prospective, non-interventional descriptive study. Consecutive LASIK patients diagnosed with unilateral or bilateral POE at their postoperative LASIK follow-up between January 2017 and December 2022 were entered into the study. Recruited patients underwent VARIO Topolyzer imaging, with the HOA excimer corneal ablation map generated at the time of POE diagnosis. This study did not reimage on subsequent visits nor look at the impact of POE progression longitudinally. A sample size of 20 patients was targeted. The target sample size of 20 patients was based on several factors, including feasibility within the study period and availability of patients diagnosed with POE during the specified timeframe. To account for the possibility of poor quality or artifactual imaging, an additional five POE patients were recruited. POE diagnosis was determined using the same previously published standardized criteria to identify POE [5, 13]. These measures included topographical changes consistent with POE together with a decrease in UDVA and/or CDVA and/or a decrease in quality of vision subjectively described by the patient as causing new or worsening haloes, glare, ghosting, or shadowing. In addition to a complete slit-lamp examination, Orbscan topography (Bausch & Lomb, Claremont, CA, USA), and corneal epithelium thickness map imaging (iVue, Optovue, Fremont, CA, USA) were taken. The cases were sent to a consultation group where a highly experienced group of five surgeons diagnosed POE. The rationale for using multiple diagnostic criteria with the consulting group of surgeons was to validate objective assessments that confirm the POE diagnosis. There were no exclusion criteria based on POE severity stage. The study was approved by the Ethics Review Board of the Canadian Ophthalmic Research Centre and fulfilled all principles of the Declaration of Helsinki. Written informed consent for anonymized data use was received from all patients.

### Topography acquisition and analysis of corneal HOA ablation map

Contoura HOA ablation map images were produced using four to eight corneal topographies acquired with the WaveLight® Topolyzer™ VARIO (Alcon) at a 6.5 mm optical zone, as previously described [8, 9, 12, 14]. The HOA ablation map scale is normalized to the deepest ablation value, resulting in each map having an identical range of colors from green to purple, irrespective of ablation depth [12]. Green and yellow colors are indicative of a shallower ablation, while red and purple colors are indicative of a deeper ablation. Deep and shallow ablation areas were identified and classified as distinct *ablation islands* [12]. Ablation islands inside and outside the 3.5 mm diameter zone were termed as paracentral and peripheral, respectively [12].

HOA Ablation maps were imported in MATLAB R2023a (MathWorks, Natick, MA, USA) and quantitatively characterized with the Image Processing Toolbox™ (MATLAB), as in our previous proof-of-concept study [12]. In our current POE eyes, we observed up to 4 recurring ablation islands on the HOA ablation map, similar to what we had seen in KC eyes [12]. For each island, the centroid, distance from the center, position (between 0 and 360°), orientation (between 0 and 180°), circularity, diameter, area, and maximal ablation depth were assessed, as described previously (Supplemental Figure A) [12]. To account for enantiomorphism, the mirror-symmetry between the right and left eyes, the orientation of all left eyes (OS) was flipped along the vertical axis during the analysis. This ensured that the generated figures and calculations accurately represented the spatial distribution of ablation islands, allowing for a comprehensive assessment of their locations and avoiding any potential misinterpretation due to asymmetric presentation between OD and OS eyes.

### Data and statistical analysis

Statistical analyses were conducted in MATLAB R2023a. Feature means, standard deviations (SD), and range were calculated after confirming that all reported variables were normally distributed with Kolmogorov–Smirnov tests. The coefficient of variation (CV) was calculated as SD divided by the mean and used to assess reproducibility of identified features Relationships between continuous variables were assessed by Pearson correlation coefficient (R-value). The significance level was set at *P* < 0.05.

## Results

**Fig. 1:**
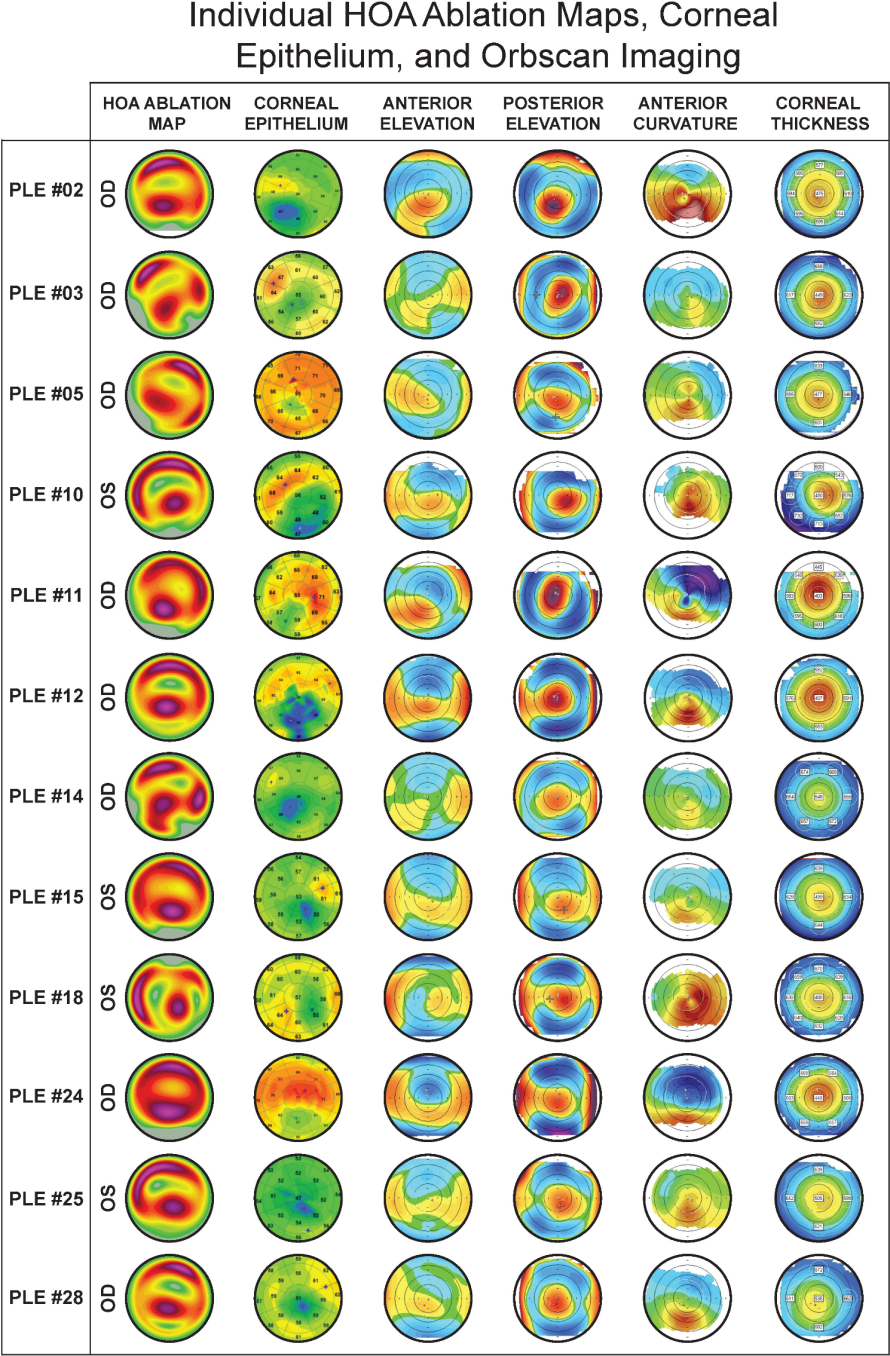
Individual higher-order aberration ablation maps, epithelium thickness profiles, and Orbscan imaging data (4 Maps – Refractive view)

A total of 33 eyes were enrolled from 25 patients. A total of five eyes were excluded from the study due to poor quality or artifactual Topolyzer VARIO scans. Namely, 3 eyes were excluded based on significant missing data (shadow from the eyelids, eyelashes, nose, or dry tear film), 1 eye was excluded for having less than 90% percentage of data obtained (analyzed area) in the 6.5-mm zone, and 1 eye was excluded for having a low median of absolute deviation variability score below 0.10. The resultant 28 eyes from 22 patients were included for analysis. The patients’ average age was 37 ± 10 years (range: 22 to 62 years). Orbscan imaging data (4 Maps – Refractive view) revealed findings consistent with POE (Fig. 1). Corneal epithelium profiles were abnormal in all eyes, showing thinning inferotemporally, superioperipheral thickening, or a partial characteristic donut pattern (Fig. 1). The topographic and epithelial OCT findings were used to confirm POE by a group of expert surgeons. The POE grade, based on the magnitude of inferior steepening, is reported in Supplemental Table A. 4 eyes had less than 2.00 D of steepening, 10 eyes had between 2.00 and 4.00 D of steepening, 4 eyes had between 4.00 and 6.00 D of steepening, 6 eyes had between 6.00 and 8.00 D of steepening, 2 eyes had between 8.00 and 10.00 D of steepening, 1 eye had between 10.00 and 12.00 D of steepening, and 1 eye had 12.00 D or move. See Supplemental Table A for additional individual Orbscan indices and group data.

### Characterization and reproducibility of 4 ablation Islands on the corneal HOA ablation map

**Fig. 2:**
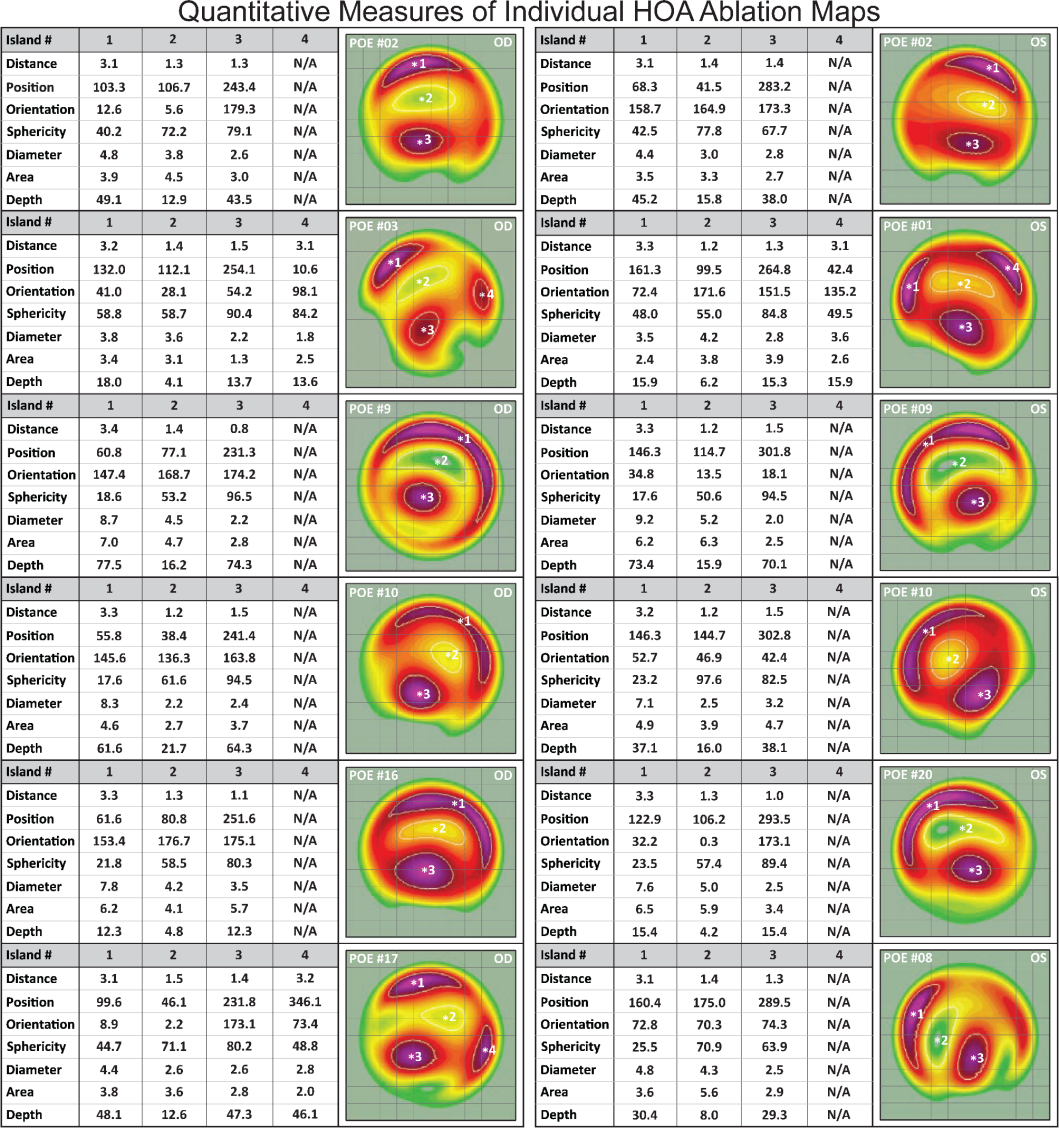
Representative HOA ablation maps. The yellow/green colors on each HOA map indicate shallower ablation values, while the red/purple colors indicate deeper ablation. The colors of each map are normalized to their deepest ablation values. Every HOA map featured one or two red/purple peripheral ablation crescents (islands 1 and 4), a yellow/green paracentral superior ablation island (island #2), and a red/purple paracentral inferior ablation island (island #3). We quantitatively characterized the HOA ablation map islands based on their distance from center, positioning, orientation, circularity, diameter, area, and depth

Representative individual POE HOA ablation maps are shown in Fig. 2 along with the characteristics of all ablation islands (methods described in Supplemental Figure A. In each HOA ablation map, we identified one (in 17/28 eyes: 60.7%) or two (in 11/28 eyes: 39.3%) red / purple deep peripheral crescent-shaped ablation islands (identified as Island #1 and #4; Fig. 2). 92.9% of the deep peripheral crescents were found in the superior quadrant, and 56.4% were oriented nasally. We also identified a yellow/green shallow paracentral superior ablation island (identified as Island #2 in Fig. 2), located in the superior quadrant in all 28 eyes (100%) as well as nasally in 19 eyes (67.9%), and a deep red/purple paracentral inferior ablation island (identified as Island #3 in Fig. 2), in the inferior quadrant in all 28 eyes (100%), as well as temporally in 26 eyes (92.9%).

Both the yellow/green paracentral superior ablation islands and the red/purple paracentral inferior ablation islands were elliptical in shape (Circularity: 69.4 ± 9.2% and 74.9 ± 14.0%; Table 1), of small diameter (3.3 ± 1.0 mm and 2.7 ± 1.0 mm), and of similar area (3.8 ± 1.6 mm^2^ and 3.4 ± 1.7 mm^2^). Due to their crescent-like shapes, the red/purple peripheral superior crescents had much smaller circularity indices (38.6 ± 16.5%) and were larger in area and longer in diameter.

**Table 1:** Mean and range of descriptors of the four HOA ablation profile islands identified in POE eyes

Descriptors	Ablation island #1		Ablation island #2		Ablation island #3			Ablation island #4	
	Mean ± SD (CV %)	Range	Mean ± SD (CV %)	Range	Mean ± SD (CV %)		Range	Mean ± SD (CV %)	Range
**Distance (mm)**	3.2 ± 0.1 (3.9%)	2.8 to 3.4	1.4 ± 0.2 (15.5%)	1.0 to 1.8	1.4 ± 0.4 (30.1%)		0.8 to 2.8	3.2 ± 0.2 (5.0%)	2.9 to 3.5
**Position (°)**	90.1 ± 65.9 (73.2%)	1.5 to 357.9	82.7 ± 47.3 (57.2%)	5.0 to 172.5	249.3 ± 17.3 (6.9%)		213.2 to 294.1	137.0 ± 120.3 (87.8%)	9.1 to 352.0
**Orientation (°)**	88.8 ± 58.1 (65.4%)	8.9 to 166.6	104.7 ± 68.4 (65.3%)	2.2 to 179.8	101.9 ± 67.7 (66.4%)		6.2 to 179.3	78.0 ± 24.6 (31.5%)	37.8 to 112.1
**Circularity (%)**	38.6 ± 16.5 (42.7%)	12.4 to 71.0	69.4 ± 9.2 (12.9%)	48.4 to 97.6	74.9 ± 14.0 (18.7%)		47.7 to 96.5	56.1 ± 15.5 (27.6%)	32.8 to 84.2
**Diameter (mm)**	5.0 ± 1.9 (37.7%)	2.5 to 9.2	3.3 ± 1.0 (31.6%)	1.2 to 5.2	2.7 ± 1.0 (35.9%)		1.8 to 6.9	2.6 ± 0.6 (22.8%)	1.8 to 3.6
**Area (mm** ^**2**^ **)**	3.9 ± 1.4 (35.3%)	1.6 to 7.0	3.8 ± 1.6 (42.3%)	0.5 to 7.4	3.4 ± 1.7 (50.7%)		1.4 to 10.0	1.7 ± 0.7 (42.6%)	0.7 to 2.9
**Depth (µm)**	34.8 ± 18.9 (54.4%)	12.3 to 77.5	11.5 ± 6.9 (60.5%)	3.6 to 25.5	32.5 ± 18.8 (50.7%)		12.1 to 74.3	23.4 ± 12.6 (51.7%)	12.6 to 46.1

Ablation island #1 = deep peripheral superior crescent; Ablation island #2 = shallow paracentral superior ablation island; Ablation island #3 = deep paracentral inferior ablation island; Ablation island #4 = deep peripheral crescent, only applicable to cases presenting with two deep peripheral crescents; CV = Coefficient of variation, POE = Postoperative LASIK ectasia

**Fig. 3:**
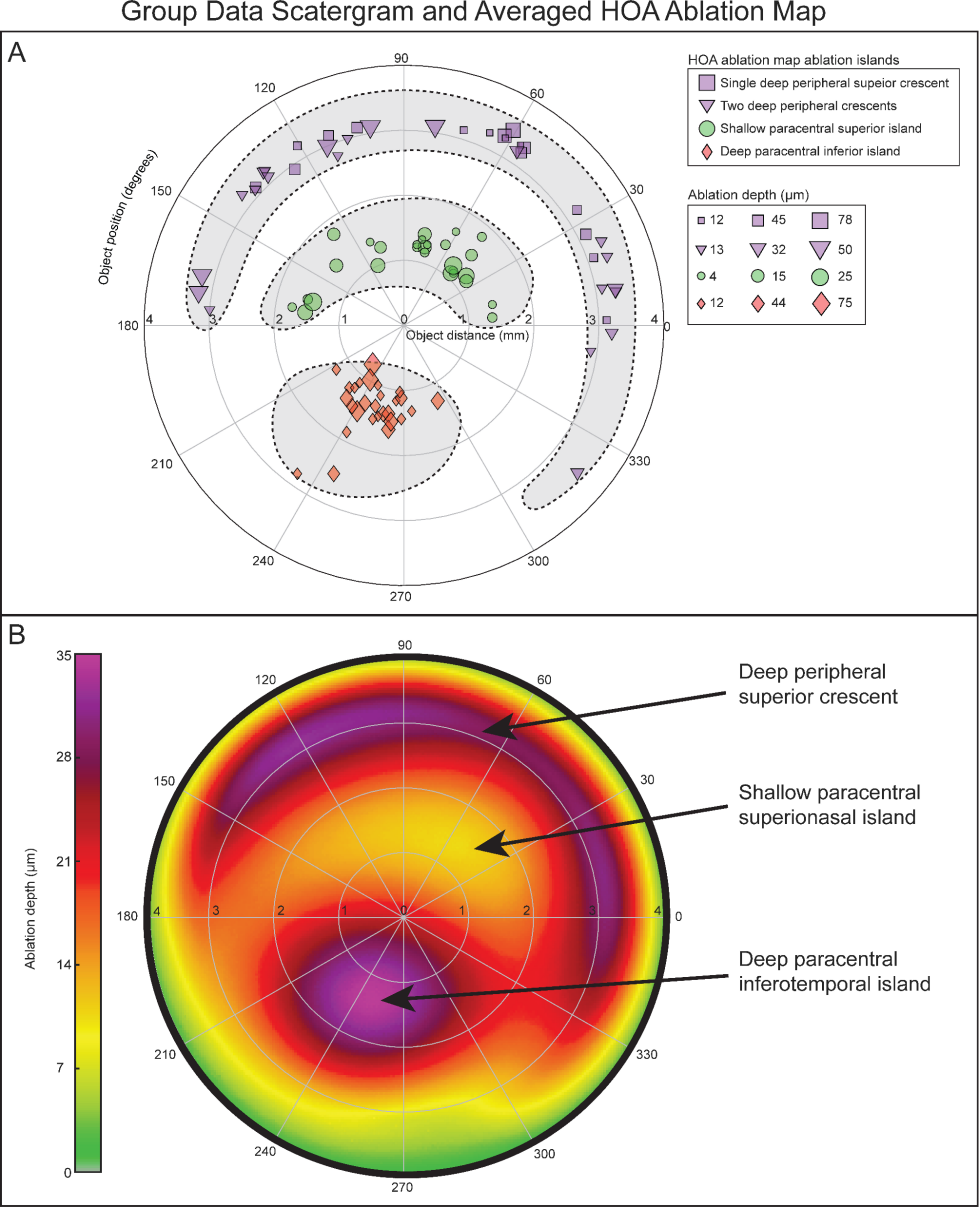
(**A**) Group data of every ablation island. The distance from center, the angular position, and the ablation depth of the paracentral superior ablation island (island #2; green circles), the red/purple paracentral inferior ablation island (Island #3; red diamonds) and the deep peripheral ablation crescents (Island #1 and Island #4; purple markers) are shown for all 28 POE eyes (**B**) Composite HOA ablation map obtained by averaging the HOA ablation map of all 28 postoperative ectasia cases

Group data reveals that the paracentral superior ablation island (Fig. 3A; green circles) and the red/purple paracentral inferior ablation island (Fig. 3A; red diamonds) were reproducible in terms of distance from center (1.4 ± 0.2 mm; CV: 15.5% and 1.4 ± 0.4 mm; CV: 30.1%). The angular position of the superior ablation island was overall superionasal (82.7 ± 47.3°) with a high variability (CV: 57.2%), while the inferior ablation island position was overall inferotemporal (249.3 ± 17.3°) and highly reproducible (CV: 6.9%). The ablation depth of the superior and inferior deep ablation island varied significantly (11.5 ± 6.9 μm; CV: 60.5% and 32.5 ± 18.8 μm; CV: 50.7%). The distance from the center of the superior peripheral ablation crescents (Fig. 3A, purple markers) was highly reproducible (3.2 ± 0.1 mm; CV: 3.9%). Peripheral crescents had a superior angular position (90.1 ± 65.9°; CV: 73.2%) and a large range of depth (34.8 ± 18.9 μm; CV: 54.4%). The data and CV for all ablation islands can be found in Table 1; Fig. 3B shows the overall shapes using the average of all 28 HOA ablation maps.

Very strong correlations were found between the depth of the deepest peripheral crescents and the depth of the paracentral inferior ablation islands (*R = 0.99*; *P* < 0.0001) and between the depths of the two paracentral ablation islands (*R = 0.83*; *P* < 0.0001). The position of the paracentral ellipses was not statistically correlated to the angular position of the peripheral crescents (*P* > 0.05).

### Correlations between ablation island features and Orbscan indices

**Fig. 4:**
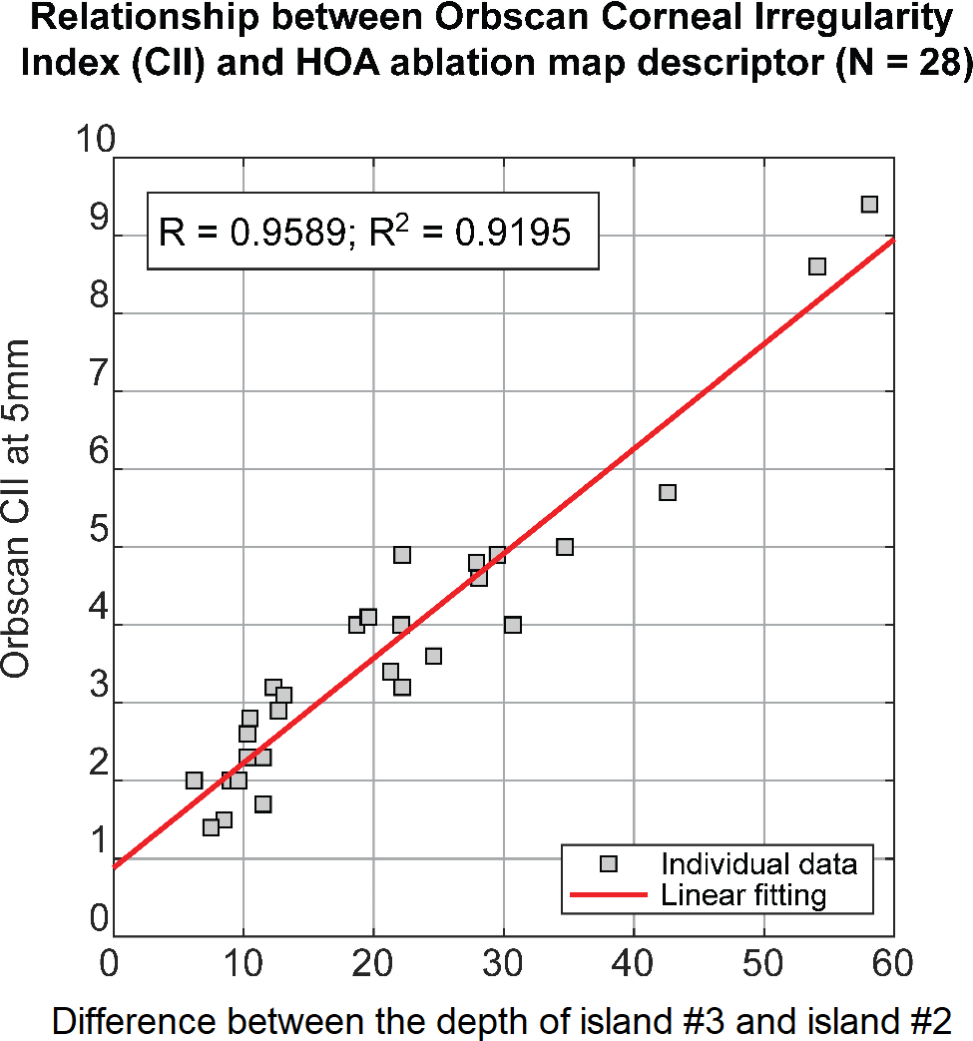
Linear relationship between Orbscan Corneal Irregularity Index (CII) at 5 mm and the difference between the ablation depth of island #3 and island #2

We examined whether the ablation depth parameter correlated with POE severity. A Pearson correlation was calculated between each ablation island depth and 8 different Orbscan indices (Kmax, Steepest K, SimK, CII(3 mm), CII(5 mm), Steepening(5 mm), CCT, and Post. Elevation: Table 2) are reported. Very strong correlations were found between the corneal irregularity index (CII at 5 mm) and the depth of the peripheral crescents (*R = 0.92*; *P* < 0.0001), between the corneal irregularity index and the ablation depth of the paracentral inferotemporal island (*R = 0.93*; *P* < 0.0001), between the corneal irregularity index and the difference of ablation depth between the deep paracentral inferotemporal island and the shallow paracentral superionasal island (*R = 0.96*; *P* < 0.0001; Fig. 4), and between the posterior elevation (Post. Elevation) and the ablation depth difference between the deep paracentral inferior island and the shallow paracentral superior island (*R = 0.84*; *P* < 0.0001). Additional statistically significant correlations are reported in Table 2.

**Table 2:** Pearson correlation between ablation depth and Orbscan Indices

Parameters	Depth of island #1/4	Depth of island #2	Depth of island #3	Δ islands #3 and #2
**Kmax**	**0.4420 (0.0185)**	-0.07 (0.7356)	**0.40 (0.0344)**	**0.59 (0.0009)**
**Steepest K**	0.24 (0.2278)	0.10 (0.6295)	0.17 (0.3881)	0.19 (0.3413)
**SimK**	**0.75 (< 0.0001)**	0.36 (0.0625)	**0.74 (< 0.0001)**	**0.84 (< 0.0001)**
**CII (3 mm)**	**0.90 (< 0.0001)**	**0.62 (0.0005)**	**0.91 (< 0.0001)**	**0.95 (< 0.0001)**
**CII (5 mm)**	**0.92 (< 0.0001)**	**0.65 (0.0002)**	**0.93 (< 0.0001)**	**0.96 (< 0.0001)**
**Steepening (5 mm)**	**0.76 (< 0.0001)**	**0.48 (0.0095)**	**0.76 (< 0.0001)**	**0.80 (< 0.0001)**
**CCT**	-0.20 (0.3080)	-0.20 (0.3103)	-0.21 (0.2942)	**-0.18 (0.3506)**
**Post. Elevation**	**0.71 (< 0.0001)**	0.37 (0. 0542)	**0.74 (< 0.0001)**	**0.84 (< 0.0001)**

Values reported as Pearson correlation coefficient R. P-values are reported in brackets. Values in bold indicate *P* < 0.05. Island #1/4 = deep peripheral crescents; Island #2 = shallow paracentral superior ablation island; Island #3 = deep paracentral inferior ablation island; CCT = Central Corneal Thickness; CII = Corneal Irregularity Index; Kmax = maximal keratometry; Post. = Posterior; SimK = Simulated keratometry (anterior); Δ = Difference between the two islands

## Discussion

By combining the anterior corneal HOA Zernike coefficients (C6 through C27) from Topolyzer VARIO high-resolution scans, the WaveLight Contoura planning software produces an anterior corneal HOA ablation map that is eye-specific. The map is used to differentially ablate small amounts of irregular corneal tissue within the spherocylindrical ablation [12]. This HOA ablation map illustrates the shapes, locations, and depths of excimer laser tissue removal patterns used to correct anterior corneal surface HOA wavefront errors [12]. Unlike Zernike whole-eye HOA values, these maps provide additional information related to position, eccentricity, depth, orientation, and shape of anterior corneal HOAs. A pilot and proof-of-concept study demonstrated the usability of the corneal HOA ablation map in characterizing keratoconus eyes of grade 2 and above [12]. This study is the first to analyze the HOA ablation map in POE eyes and to objectively quantify the graphical representation of anterior corneal HOAs. Seven objective descriptors from 4 ablation islands were derived. These new metrics can now be used to describe features seen in POE eyes and would be applicable to the HOA ablation maps of any topography-guided platform.

In POE eyes, recurring ablation islands were consistently seen on the corneal HOA ablation maps. These consisted of two elliptical paracentral mirror image ablation islands of similar circularity and area, with a deeper inferior island (inferior quadrant in 100% of eyes and temporally in 92.9% of eyes) and its shallow corresponding superior island (superior quadrant in 100% of eyes, and nasally in 67.9% of eyes). The deep paracentral inferior island had high reproducibility in angular position. The distance from center was also reproducible. Both paracentral ablation islands were always within a 3.4 mm diameter central ring (maximum 1.7 mm radius from center). One or two superior crescents of varying lengths were also consistently seen within the paracentral 6-7 mm diameter ring, positioned in the superior quadrant in 93% of cases and nasally in 56% of cases. The above-reported distances are those obtained with a 6.5 mm optical zone size, used in all eyes in the current study.

The deeper paracentral inferior ellipse visually corresponded to both anterior and posterior topography/tomography elevation peaks and the corneal thinnest spot. Our previous study in keratoconus showed that, on average, the inferotemporal deeper island was nearly identical in distance to the cone apex center seen on topography elevation maps [12]. Similar findings were found in the current study of POE eyes. The inferior angular position of the deep paracentral ablation island (249.3 ± 17.3°) was consistent with the POE cone position and with the previously reported KC cone apex position [12]. Based on our current and previous findings, the inferotemporal “hot spot” can be reliably identified as the apical cone. Our former study characterizing HOA maps in keratoconus eyes revealed a similar pattern of superior peripheral crescents, a shallow superionasal ablation island, and a deep inferotemporal ablation island [12].

The deep and shallow paracentral ellipses were positioned as directly opposing each other in all eyes. These islands represent the differential corneal ablation pattern that is required to correct the comatic aberrations that are caused by a localized decentered corneal elevation – seen in POE. Likewise, the peripheral crescent ablation contributes to comatic correction by causing a relative elevation more centrally with a further peripheral ablation superiorly. This explains why in eyes with coma-dominated optics, the depth of the paracentral islands and peripheral crescents have previously been found to be highly correlated [12]. Coma was always the dominant aberration in all POE eyes, regardless of concomitant spherical aberration and trefoil, like KC eyes [12].

Regardless of where the decentered POE cone was located, the characteristic comatic ablation pattern was identified and was identical to the pattern found in KC eyes [12]. Anterior wavefront aberrometry maps have shown similar characteristic coma patterns in POE and KC eyes, with a familiar superior red and inferior blue opposing ellipses [15–19]. Patterns derived from wavefront maps lack details regarding the location and depth of the irregularity causing the aberration on the cornea. By measuring both the ablation depth and the location of comatic aberrations on the cornea, the corneal HOA ablation map provides novel graphical and quantitative information that are anatomically linked to the corneal surface [12]. While the use of wavefront aberration metrics is not new, the novelty of our work lies in the characterization of the HOA ablation map and its potential value as a diagnostic and monitoring tool for POE. Identifying the highly recognizable patterns in clinical practice - seen here in all 28 POE eyes - should lead to further investigation and OCT epithelial imaging to confirm the POE diagnosis. Comparison group studies with specificity and sensitivity analyses would add value in confirming the HOA ablation map’s role in identifying POE eyes.

Previous studies have used anterior corneal coma Zernike coefficients to diagnose KC and monitor its progression [15, 19–22]. However, these Zernike metrics have not been extensively studied and currently have insufficient sensitivity and specificity in early KC and POE. While the HOA ablation map is derived from Zernike coefficients, the ablation map provides additional graphical and depth data regarding HOAs. This more comprehensive graphical representation may be found to improve diagnostic accuracy beyond Zernike coefficients.

The maximal depth in the HOA ablation map has been shown to be directly and highly correlated with the amount of anterior corneal HOAs, with total coma having the highest correlation [23]. A previous study showed that the maximal HOA ablation map depth in normal eyes, using a 6.5 mm optical zone, was 8.02 ± 3.00 μm, compared to 34.85 ± 18.9 μm in the current study’s POE eyes, which is 4.3-fold higher [8]. A deeper value of 74.5 μm was seen in KC eyes which had greater severity of Grade 2 and above [12]. In addition, the ablation depth had a larger coefficient of variation than in KC eyes for all ablation islands, which reflects the varying severity of POE cases included in the current study. Pearson correlations revealed Orbscan Sim K, CII(3 mm), CII(5 mm), and post elevation as having very strong correlations with the maximum ablation depth of the paracentral inferotemporal ablation island. The relationship between the Orbscan corneal irregularity index (CII) and the difference between the deep paracentral inferior island (island #3) and the shallow paracentral superior island (island #2) led to the highest correlation (R = 0.96). (Fig. 4).

As the difference between the ablation depth of the deep and shallow ablation islands increases, the amount of coma reflected by corneal irregularity is increasing. The depth difference of the two islands may therefore have the potential to serve as a novel metric for quantifying POE severity. However, it is essential to compare this indicator with traditional standards to draw meaningful conclusions. Further studies to determine the best statistical threshold to classify normal vs. ectatic corneas could prove useful.

Schäffeler & Kohnen previously reported that coma aberrations were significantly correlated with POE severity [24]. Padmanabhan and colleagues also found coma aberrations and spherical aberrations to be significantly correlated with the grade of POE [25]. Delgado et al. previously reported a high correlation between coma aberration and KC severity (R = 0.60) [26]. The current POE study found much higher correlations (R = 0.96), even higher than our KC study (R = -0.74). High correlations were also found between the inferior paracentral ablation island’s maximum depth and Orbscan grading indices (Kmax, SimK, CII [3 and 5 mm], steepening, and posterior elevation). These high correlations suggest that the HOA ablation map may have the potential to increase the sensitivity and specificity of POE diagnosis and grading compared to individual corneal Zernike coefficients alone.

Even though this case series is the first to describe the HOA ablation map in POE eyes, future studies with a larger sample size and a comparative group will be needed to evaluate the diagnostic power of these novel descriptors. Determining if other clinical conditions create a similar pattern, quantifying differences, and establishing discriminating values would be useful. The question of how frequently healthy non-POE LASIK eyes have the same HOA ablation map pattern as our POE cohort, yet never developing ectasia, has not been investigated. Determining the sensitivity and specificity of the HOA ablation map would be the subject of future studies. Future studies will aim to confirm that the described POE features found in the current study are specific to POE by comparing the HOA ablation map of POE eyes to a group of normal postoperative corneas, a group of suspect POE corneas and a group of definitive POE corneas. While our study focused on characterizing the HOA ablation map in POE eyes, we acknowledge the potential benefits of incorporating VARIO Topolyzer imaging as a routine component of postoperative follow-ups. This would allow for the assessment of preoperative and postoperative corneal characteristics, enabling a more comprehensive evaluation of the predictive value of VARIO Topolyzer imaging for early-stage POE. Furthermore, investigating the long-term outcomes and comparing the incidence of POE in treated versus untreated eyes would help determine whether LASIK indeed induces ectasia or simply accelerates its presentation. While a longitudinal study would be necessary to fully address the question of causality, the purpose of our study was not to establish causality, but rather characterize the maps in POE eyes. Comparison to existing technologies for early keratoconus and POE detection and disease progression also warrants further investigation. While the specific characteristics of the HOA ablation map might differ across topography-guided platforms due to various proprietary algorithms and tweaks, the underlying concept of analyzing HOA ablation patterns remains relevant. We encourage further investigations and collaborations involving different platforms (e.g., from Zeiss, Nidek, IVis, or Schwind) to assess the generalizability and transferability of the HOA ablation map and enhance its clinical utility. In this preliminary study, our focus was on using and characterizing the raw original HOA ablation maps without normalization. Future studies might explore the potential benefits of transforming and/or normalizing the HOA ablation maps to a specific position or depth, such as that of the peripheral superior crescents or other islands. The above-mentioned detailed investigations were not the goal of the current pilot study in POE eyes.

## Conclusions

In summary, this study qualitatively and quantitively characterizes reproducible features of the HOA ablation map in POE eyes and adds novel objective measures of POE corneal irregularities. Like KC eyes, POE eyes with an inferiorly displaced cone produce a unique and highly recognizable pattern on the HOA ablation map. Namely, two paracentral ablation islands in direct mirror-like opposition to each other, one shallower superior and one deeper inferior, which are accompanied by longer peripheral, mostly superior, crescents of ablation. The deep inferior ablation island is coincident with the topographical POE cone, and the depth difference between the shallow and deep paracentral islands strongly correlates to Corneal Irregularity Indices (CII) at 3 and 5 mm. Further studies should be conducted to determine cut-off values for separating normal postoperative eyes from clinical POE with sensitivity and specificity. The corneal HOA ablation map can potentially yield new information that can be used to diagnose, grade, and monitor POE progression both before and after corneal crosslinking.

### Electronic supplementary material

Below is the link to the electronic supplementary material.

Supplementary Material 1

Supplementary Material 2

## Data Availability

All data generated or analysed during this study are included in this published article and its supplementary information files.

## List of abbreviations

CDVA: Corrected Distance Visual Acuity
HOA: Higher-order aberration
KC: Keratoconus
LASIK: Laser-Assisted in Situ Keratomileusis
LRS: Laser Refractive Surgery
POE: Postoperative ectasia
PRK: Photorefractive Keratectomy
UDVA: Uncorrected Distance Visual Acuity
