# Supplementary material for: Characterization of postoperative LASIK ectasia features on higher-order aberration excimer ablation maps
Source: BMC Ophthalmol. 2023 Dec 20;23:517. doi: 10.1186/s12886-023-03263-y (PMC10734092; doi:10.1186/s12886-023-03263-y)
Supplement: Supplementary file 1 — Supplementary Material 1 [file 12886_2023_3263_MOESM1_ESM.pdf]

Supplemental Figure A

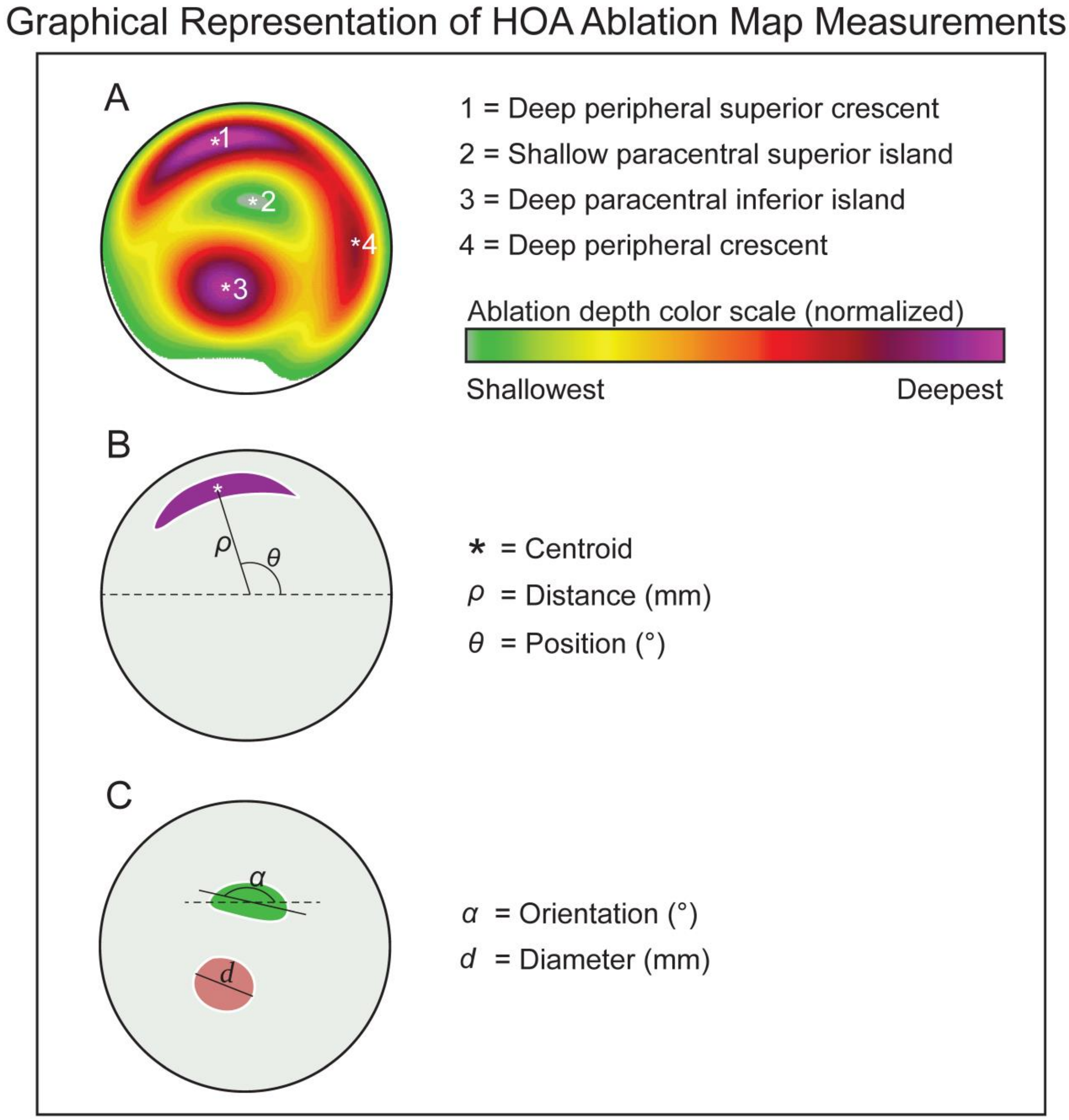

Supplemental Figure A. A representative HOA ablation map shows four distinct ablation islands in POE eyes **(A)**. The ablation islands' *distance from center* and *position* features are represented graphically in **(B)**. The *distance from center* (ρ) is expressed as the radius (in mm) between the centroid of any ablation island and the center of the HOA ablation map. Island centroids are denoted by white asterisks (*). In order to determine the orientation (θ) of an ablation island, we calculate the angle (between 0 and 360º) formed between its centroid and the dotted line (abscissa). **(C)** The *diameter* and *orientation* features of an ablation island are illustrated graphically. The *diameter* feature of an ablation island was defined as the length of its major axis (d). The ablation island orientation (α) is defined as the angle formed between the longest diagonal of the ablation island (called the major axis in geometry) and the dotted horizontal line passing by the island's centroid. We also calculated *circularity* and the *area* (mm^2) of each ablation island. The *area* was calculated by multiplying the number of pixels by the area of one pixel. The *circularity* of an ablation island was defined as 4π times the area divided by the square of the perimeter. Circularity is equal to 1.0 for a perfect circle, and it is smaller than 1.0 for an ellipse or a crescent shape.
