# Supplementary material for: Characterization of postoperative LASIK ectasia features on higher-order aberration excimer ablation maps
Source: BMC Ophthalmol. 2023 Dec 20;23:517. doi: 10.1186/s12886-023-03263-y (PMC10734092; doi:10.1186/s12886-023-03263-y)
Supplement: Supplementary file 2 — Supplementary Material 2 [file 12886_2023_3263_MOESM2_ESM.docx]

**Supplemental Table A: Description of Orbscan IIz indices**

| **Case ID** | **Kmax** | **Steepest K** | **SimK** | **CII 3mm** | **CII 5mm** | **Inf.-Sup. Steepening** | **CCT** | **Post. Elevation** | **Max Post. From Center.** |
| --- | --- | --- | --- | --- | --- | --- | --- | --- | --- |
| POE #01OS | 45.1 | 46.5 | 0.4 | 1.9 | 2.0 | 2.5 | 454.0 | 54.0 | 1.0 |
| POE #02OD | 47.0 | 50.9 | 3.5 | 4.4 | 4.0 | 8.1 | 487.0 | 85.0 | 1.3 |
| POE #02OS | 45.2 | 48.5 | 2.2 | 3.3 | 3.2 | 5.7 | 512.0 | 59.0 | 1.6 |
| POE #03OD | 42.8 | 44.2 | 1.7 | 1.9 | 2.0 | 2.7 | 470.0 | 74.0 | 0.4 |
| POE #04OD | 43.9 | 44.9 | 2.0 | 1.5 | 1.7 | 1.0 | 500.0 | 44.0 | 0.3 |
| POE #05OD | 44.5 | 46.5 | 2.2 | 3.0 | 2.8 | 3.1 | 492.0 | 51.0 | 0.8 |
| POE #06OD | 42.4 | 45.5 | 1.3 | 3.5 | 3.2 | 5.0 | 458.0 | 57.0 | 0.6 |
| POE #07OD | 44.4 | 43.7 | 1.1 | 2.1 | 2.0 | 1.5 | 437.0 | 63.0 | 0.6 |
| POE #08OS | 42.2 | 45.3 | 2.3 | 3.6 | 3.4 | 3.1 | 434.0 | 55.0 | 0.9 |
| POE #09OD | 54.0 | 44.3 | 4.8 | 8.8 | 9.4 | 12.0 | 466.0 | 139.0 | 0.8 |
| POE #09OS | 53.3 | 47.9 | 5.6 | 8.5 | 8.6 | 11.0 | 480.0 | 133.0 | 1.1 |
| POE #10OD | 42.2 | 46.7 | 3.6 | 5.4 | 5.7 | 6.4 | 424.0 | 83.0 | 0.5 |
| POE #10OS | 40.9 | 43.9 | 1.6 | 4.5 | 4.0 | 2.4 | 436.0 | 87.0 | 0.7 |
| POE #11OD | 42.6 | 43.8 | 1.7 | 2.1 | 2.3 | 2.7 | 490.0 | 57.0 | 1.1 |
| POE #12OD | 42.9 | 44.3 | 0.7 | 1.7 | 1.5 | 2.2 | 561.0 | 39.0 | 0.6 |
| POE #13OD | 42.9 | 45.2 | 0.8 | 2.9 | 2.6 | 1.5 | 545.0 | 42.0 | 0.6 |
| POE #13OS | 44.1 | 48.5 | 1.4 | 4.8 | 4.6 | 6.0 | 510.0 | 62.0 | 1.1 |
| POE #14OD | 45.1 | 48.5 | 1.4 | 3.1 | 2.9 | 3.2 | 490.0 | 44.0 | 0.6 |
| POE #15OS | 46.5 | 48.3 | 2.0 | 3.9 | 4.0 | 1.9 | 505.0 | 56.0 | 0.8 |
| POE #16OD | 42.9 | 43.8 | 0.4 | 1.2 | 1.4 | 8.1 | 439.0 | 59.0 | 0.7 |
| POE #17OD | 49.5 | 45.1 | 5.8 | 4.8 | 5.0 | 6.5 | 474.0 | 96.0 | 1.3 |
| POE #18OD | 43.3 | 45.3 | 2.9 | 3.4 | 3.6 | 6.0 | 368.0 | 41.0 | 1.0 |
| POE #19OD | 41.9 | 45.8 | 1.5 | 4.4 | 4.9 | 3.5 | 423.0 | 54.0 | 1.3 |
| POE #19OS | 42.5 | 47.3 | 1.2 | 3.4 | 4.1 | 5.5 | 495.0 | 40.0 | 0.8 |
| POE #20OS | 44.5 | 46.4 | 1.3 | 2.2 | 2.3 | 3.0 | 517.0 | 44.0 | 0.8 |
| POE #21OD | 40.8 | 43.7 | 3.2 | 4.8 | 4.9 | 6.2 | 503.0 | 79.0 | 1.7 |
| POE #21OS | 39.2 | 44.4 | 1.4 | 4.8 | 4.8 | 7.1 | 520.0 | 70.0 | 1.8 |
| POE #22OD | 43.3 | 47.5 | 1.3 | 3.5 | 3.1 | 5.6 | 535.0 | 43.0 | 0.6 |
| **Average** | **44.3 ± 3.3** | **46.0 ± 1,9** | **2.1 ± 1.4** | **3.7 ± 1.8** | **3.7 ± 1.9** | **4.8 ± 2.8** | **479.5 ± 41.2** | **64.6 ± 25.5** | **0.9 ± 0.4** |

CCT = central corneal thickness; CII = corneal irregularity index; Inf = inferior; K = keratometry; Kmax = maximal keratometry; Post = Posterior;

SimK = simulated keratometry; Sup = Superior.
